# 

**Published:** 1881-12

**Authors:** 


### OHIO STATE SOCIETY.

The Sixteenth Annual Meeting of the Ohio State Dental Society will be held in the Board of Trade Rooms, City of Columbus, on Dec. 7, 1881, commencing at 10 o'clock, A. M., and continue its sessions three days, adjourning Friday afternoon. The indications are that it will be a large and interesting meeting. Let everybody be there.

Alex

527

### CONTENTS VOL. XXXV.

A Cup of Tea .................... 26
Anæsthesia—The Law............ 44
About Coin...................... 85
Alumni Meetings..................
A German View of the Qualifications of a Physician...... 126
A New Journal.................... 132
Alveolar Abscess................. 133
Absorbent Cotton................. 166
Association ....................... 174
A Proper Recognition............ 176
Annual Meeting .................. 176
Anatomical Material............. 205
Association.....................218–220
Artificial Crowns................. 221
Another Case of Maternal Impressions with Singular Malformation.................. 248
Anatomical Charts................ 260
Alabama Dental Association..... 266
Alveolar Ulceration Distinguished from Alveolar Abscess... 267
An Ideal.......................... 279
American Dental Association.... 312
Annual Meeting of the Illinois State Dental Society......... 337
Alumni Matters................... 352
A New Arm Rest.................. 398
Antiseptic Treatment of Alveolar Abscess.................. 399
An Interesting Case............... 425
A Novel Case..................... 438
A Directory....................... 442
Address ........................... 443
Anæsthesia—Nitrous Oxide...... 458
A Hitherto Undescribed Disease of Children. ................... 471
A Plea for General Education on Subjects of Hygiene.......... 473
Analysis of the Human Breath.. 477
A New Journal ................... 484
Action Upon the Fœtus of Medicine taken by the Mother... 518
Bacteria in the Air.............. 203
Bibliographical .................. 219
Bleeding in Tetanus............. 251
Chronic Chloral Poisoning.. ..... 51
Case of Hemorrhagic Diathesis.. 64
Concerning the Nerve Development of the Pathology of Nerves, and its Importance in Medical Instruction........ 93
Certified .......................... 132
Commencement .................. 173
Causes of Suicide................. 250
Correspondence 254-266-296-382–481
Correction ........................ 261
Change of Time.................. 264
Concerning Dental Colleges...... 314
Cholera Infantum................. 383
Cancrum Oris..................... 346
Correspondence .................. 350
Carbolic Acid Poisoning.......... 426
Claims of the Microscope......... 519
Curious Sanitary Discoveries..... 522
Dangers of Anæsthesia............ 32
Development of the Teeth, with their Arterial and Nervous Supply......................... 177
Dental Almanac................... 217
Disease of the Fifth Nerve........ 307
Deciduous Teeth.................. 379
Death from Anæsthesia........... 475
Digestive Ferment in the Fig.... 477
Dentition and Misnomer.......... 433
Deaths from Ether ............... 514
Engagement Book..............41-523
Ether and Chloroform............ 66
Electric Light good for the Eyes 71
Effects of an Over-Dose of Belladonna........................... 74
Effects of Electric Light on the Eyes............................ 200
Education ........................ 215
Editorial .......................... 351
Extracting ........................ 415

Examiners' Meeting................ 485
Elevation of the Epiglottis...... 478
Fatal Poisoning by Inhalation of Carbolic Acid.................... 515
Facial Neuralgia cured by a New Surgical Operation........... 29
Guilding.............................. 69
Go away from Home to learn the News............................ 204
Give your Titles.................... 471
Hymenial .......................... 44
Headache in School Children.... 68
Hot Water Compresses in Tetanus............................ 127
Hospital Clinics....................... 129
Howard's Method of Artificial Respiration...................... 394
Hot Water for the Heart ........ 475
Hints for Using Iodoform.......... 515
Influence of Light on Nutrition 27
Injuries to Nerves.................. 30
Is it a College?..................... 80
International Medical Congress.. 84
Iowa State Dental Society......... 88
In Memory of Dr. Jas. Taylor.... 308
International Med. Congress 311-376
Inoculation of Saliva............... 474
Longevity of the Friends........ 73
Literary Recreation.................. 137
Local Anæsthesia of the Larynx 202
Legal.................................... 213
Leeches.............................. 246
Miss. Valley Dental Society...... 43
Michigan " " ..... 131
Miss. Valley Association of Dental Surgeons........................ 154
Medical Diplomas.................... 203
Maternal Impressions..........427-479
Microscopy and Pathology........ 435
Medical Students .................... 499
Mouth Wash for Tobacco Consumers............................... 523
National Dental Association ..... 313
Natural History Notes............ 344
Neuralgia as a Warning........... 345
New Instruments for the Engine by means of which Gold Fillings can be rapidly and securely Inserted.............. 393
Northern Ohio Dental Association................................. 395
New Method of Preparing the Spinal Cord for Microscopic Sections............................ 436
New Injection Method............. 438
Nitrous Oxide......................... 483
On the Comparative Value of Sulphuric Acid and Creosote in the Treatment of Alveolar Caries ............... 8
On the Behaviour of Plaster in Setting............................. 23
Ohio State Dental Society..42-87-485
Ohio State Journal of Dental Science............................ 131
Ohio College of Dental Surgery.................................. 171
Obstetrics............................. 249
Obituary ............................... 310
Oral Electricity and the New Departure ......................... 315
On the Pathology of Acute Periostitis ............................. 428
Origin of the Nervous System... 478
Operative Dentistry................. 489
Our Medical Literature........... 503
Pacific Coast Fisheries............ 26
Persistent Priapism................ 28
Pathology of the Osseous Structure of the Teeth............... 116
Proceedings of the Iowa State Dental Society.................. 303
Prosthetic Dentistry................ 330
Physical Changes Affecting the Teeth of the Young........... 411
Pregnancy and Caries of the Teeth ............................... 476
Paresis Hæmostatic Collodion.... 478
Practical Hints on Mounting..... 512
Perfumed Carbolic Acid........... 72
Quillaia Toothwash.................. 72
Response to the Toast–Dentistry and Dental Education.... 184
Removal of Tumors without leaving marks.................. 22
Rapid Breathing as a Pain Obtunder in Minor Surgery, etc.................................... 224
Restoring Partial Loss of the Teeth with Plate................ 275
Resharpening Files................. 306
Removal of a Double-Pointed Needle from the Submaxillery Connective Tissue ..... 388
Reflex Neuralgia by Foreign Body in the Ear................. 437
Removal................................ 486
Rodent Ulcer and Epithelioma... 511
Rubber Dam Holder................. 526
Removal................................ 526

Supreme Court Decision.......... 33
Specializing.......................... 41
Some Medical Quacks ............ 55
Soft Foil.............................. 82
Synopsis of the Report of Analysis of Ohio River Water... 113
Self-Confidence an Essential Element in the Character of the Ideal or Model Dentist.. 160
Substitute for Tobacco........... 210
Science Illustrated................ 262
Special Notice...................... 265
Southern Dental Association..... 265
Surgery—Syphilitic Alterations of the Teeth.................. 386
Spasm of the Œsophagus......... 429
Solution and Suspension.......... 432
Successful Transplanting of Human Bone........................ 437
Syrup of Chloral .................. 479
Septicæmia Resulting from a Leech Bite...................... 481
The Covering of Exposed Pulps 1
The Action of Medicines......... 14
The Way of Transgressors........ 28
The Determination of Normal Vision........................... 59
The Treatment of Insomnia..... 63
The Metric System in Machine Shops................... 65
The Bogus Diploma Business in Pennsylvania .................. 67
The Use of Chlorophyl in Vegetable Growth................... 70
The Goodyear Vulcanite Co...... 78
The sad End of a Promising Life.............................. 86
Tooth Structure.................... 89
Trichinæ in a Cat.................. 129
Third Annual Meeting of the Alumni Association of the Ohio College of Dental Surgery.............................. 128
Thirty-Seventh Annual Meeting of the Miss. Valley Dental Society ........................ 142
The Education of Dentists....... 194
The Pharmacology of Chloral Hydrate.......................... 200
The Metric System................. 201
The Anæsthetic Action of Electricity ........................... 201
The Structure of Metals.......... 209
The Daily Work of the Heart.. 210
The Treatment of Cancer of the Tongue.......................... 250
The Eleventh Annual Meeting of the Wisconsin State Dental Society.................. 261
The Kentucky State Dental Society ............................. 264
To the American Dental Association ........................... 266
The Fundamental Part of Anatomy—the Bones............. 287
Tobacco ............................. 306
The Mouth........................... 307
The Study of Dental Surgery and the Means thereto............ 355
Translations......................... 391
The Hearing Patent................ 393
Taking a Bite....................... 394
The Goodyear Patent.............. 395
Tincture of Chloride of Iron...... 395
The Relation of Dentistry to Medicine.......................... 422
Translations ....................... 424
The Committee on American Med. Colleges................... 430
The Trials of the Profession...... 466
The Medicinal Use of the Tomato 472
The Ohio State Dental Society... 486
Therapeutical Requisites Necessary to the Salvation of every Dental Practitioner... 47
Treatment of Pulpless Teeth and Manner of Filling Roots..... 447
The Compensation of Expert Witnesses......................... 513
The Treatment of Pulpless Teeth 491
The Merits of Soft and Cohesive Gold as Filling Materials... 493
The Ventilation of Bed Rooms.. 516
Treatment of Nasal Catarrh...... 517
The Care of the Dentists' Hands 520
The Hippocratic Oath.............. 521
The Physician's Visiting List..... 523
The Dental Law in Ohio.......... 524
What has become of the Metric System ?......................... 62
What are high Prices ?............ 389
What Teachers to Avoid.......... 431
Whiskey and Sunstroke........... 473
What Ails the Dental Societies ? 497
Use of Tobacco...................... 343
Universal Rubber Dam Screw-Clamp........................... 525
Vitality of Infants.................. 474

### THE DENTAL COLLEGE
### OF THE
### UNIVERSITY OF MICHIGAN.

The Seventh Annual Session of this Institution will commence on the 1st of October and close on the last Wednesday in March, thus making a course of six months. The regular course of instruction commences at once, and will proceed through the term, with the customary vacation.

The students in this department will receive instructions in Anatomy, Physiology, Pathology, Chemistry, Materia Medica, Therapeutics and Surgery, from the Professors of their respective branches in the *Department of Medicine and Surgery* of the University, when lectures commence, and continue the same as with the Dental College. There will also, in addition, be a special course upon each of these branches. These courses will each embrace from ten to fifteen lectures.

### FACULTY OF THE DENTAL DEPARTMENT.

J. B. ANGELL, LL. D., - - - - - - - - Presiaent.
J. TAFT D. D. S., - Principles and Practice of Operative Dentistry.
CORYDON L. FORD, M. D., D. D. S., - - Anatomy and Physiology.
J. A. WATLING, D. D. S., - - Clinical and Mechanical Dentistry.
W. H. DORRANCE, D. D. S., - Demonstrator of Mechanical Dentistry.

### SPECIAL COURSES.

A. B. PALMER, M. D., - - - - - - Dental Pathology.
DONALD MACLEAN, M. D., - - - - - - Oral Surgery.
E. S. DUNSTER, M. D., Diseases of Women and Children with reference to the Teeth.
GEO. E. FROTHINGHAM, M. D., - - - - Dental Therapeutics.

*Geo L. Field, D. D. S , will give Special Instruction in Continuous Gum Work.*

Students should be promptly present at the College on Friday, September 30th, at 10 o'clock A. M., to make the preliminary arrangements for entering upon regular work on the morning of October 1st. Seats in the lecture room are assigned by selection to students in the order of registration on the Steward's books; and each student is expected to occupy during the session such seat as he may select. Students on arriving at Ann Arbor, should call at the Steward's office.

### CONDITIONS OF GRADUATION.

The candidate must be twenty-one years of age. He must furnish evidence of good moral character.

He must devote three years to the study of his profession. He must attend two full courses of lectures in the Dental College, or one course in some college having an equal standard of requirements, and the last one here, and we recommend that he attend three courses regularly.

He must sustain an examination satisfactory to the Faculty in all the branches taught.

A graduate of the Medical College may enter the senior class, and, if found qualified, may graduate after one year has been devoted to the study of dentistry.

### FEES AND EXPENSES.

The fees, which must be paid in advance, are as follows:

Residents of Michigan.—Matriculation fee, $10.00; annual dues, $20,00.

Non-Residents.—Matriculation, $25.00; annual fees, $25.00.

Graduation Fee.—For all alike, $10.00. The admission fee is paid but once, and entitles the student to the privileges of permanent membership in any department of the University. The annual due is paid the first year and every year thereafter while at the University.

For further particulars, address the Dean of the Dental College Ann Arbor, Michigan.

**J. TAFT, Dean.**

Apr6mo

### THE DENTAL COLLEGE
### OF THE
### UNIVERSITY OF MICHIGAN.

The Seventh Annual Session of this Institution will commence on the 1st of October and close on the last Wednesday in March, thus making a course of six months. The regular course of instruction commences at once, and will proceed through the term, with the customary vacation.

The students in this department will receive instructions in Anatomy, Physiology, Pathology, Chemistry, Materia Medica, Therapeutics and Surgery, from the Professors of their respective branches in the *Department of Medicine and Surgery* of the University, when lectures commence, and continue the same as with the Dental College. There will also, in addition, be a special course upon each of these branches. These courses will each embrace from ten to fifteen lectures.

### FACULTY OF THE DENTAL DEPARTMENT.

J. B. ANGELL, LL. D., - - - - - - - Presiaent.
J. TAFT D. D. S., - Principles and Practice of Operative Dentistry.
CORYDON L. FORD, M. D., D. D. S., - - Anatomy and Physiology.
J. A. WATLING, D. D. S., - - Clinical and Mechanical Dentistry.
W. H. DORRANCE, D. D. S., - Demonstrator of Mechanical Dentistry.

### SPECIAL COURSES.

A. B. PALMER, M. D., - - - - - - Dental Pathology.
DONALD MACLEAN, M. D., - - - - - - Oral Surgery.
E. S. DUNSTER, M. D., Diseases of Women and Children with reference to the Teeth.
GEO. E. FROTHINGHAM, M. D., - - - - Dental Therapeutics.

*Geo L. Field, D. D. S, will give Special Instruction in Continuous Gum Work.*

Students should be promptly present at the College on Friday, September 30th, at 10 o'clock A. M., to make the preliminary arrangements for entering upon regular work on the morning of October 1st. Seats in the lecture room are assigned by selection to students in the order of registration on the Steward's books; and each student is expected to occupy during the session such seat as he may select. Students on arriving at Ann Arbor, should call at the Steward's office.

### CONDITIONS OF GRADUATION.

The candidate must be twenty-one years of age. He must furnish evidence of good moral character.

He must devote three years to the study of his profession. He must attend two full courses of lectures in the Dental College, or one course in some college having an equal standard of requirements, and the last one here, and we recommend that he attend three courses regularly.

He must sustain an examination satisfactory to the Faculty in all the branches taught.

A graduate of the Medical College may enter the senior class, and, if found qualified, may graduate after one year has been devoted to the study of dentistry.

### FEES AND EXPENSES.

The fees, which must be paid in advance, are as follows:

RESIDENTS OF MICHIGAN.—Matriculation fee, $10.00; annual dues, $20,00.

NON-RESIDENTS.—Matriculation, $25.00; annual fees, $25.00.

GRADUATION FEE.—For all alike, $10.00. The admission fee is paid but once, and entitles the student to the privileges of permanent membership in any department of the University. The annual due is paid the first year and every year thereafter while at the University.

For further particulars, address the Dean of the Dental College, Ann Arbor, Michigan.

**J. TAFT, Dean.**

Apr6mo

### THE DENTAL COLLEGE

### OF THE

### UNIVERSITY OF MICHIGAN.

The Seventh Annual Session of this Institution will commence on the 1st of October and close on the last Wednesday in March, thus making a course of six months. The regular course of instruction commences at once, and will proceed through the term, with the customary vacation.

The students in this department will receive instructions in Anatomy, Physiology, Pathology, Chemistry, Materia Medica, Therapeutics and Surgery, from the Professors of their respective branches in the *Department of Medicine and Surgery* of the University, when lectures commence, and continue the same as with the Dental College. There will also, in addition, be a special course upon each of these branches. These courses will each embrace from ten to fifteen lectures.

### FACULTY OF THE DENTAL DEPARTMENT.

J. B. ANGELL, LL. D., - - - - - - - President.
J. TAFT D. D. S., - Principles and Practice of Operative Dentistry.
CORYDON L. FORD, M. D., D. D. S., - - Anatomy and Physiology.
J. A. WATLING, D. D. S., - - Clinical and Mechanical Dentistry.
W. H. DORRANCE, D. D. S., - Demonstrator of Mechanical Dentistry.

### SPECIAL COURSES.

A. B. PALMER, M. D., - - - - - - Dental Pathology.
DONALD MACLEAN, M. D., - - - - - - Oral Surgery.
E. S. DUNSTER, M. D., Diseases of Women and Children with reference to the Teeth.
GEO. E. FROTHINGHAM, M. D., - - - - Dental Therapeutics.

*Geo. L. Field, D. D. S., will give Special Instruction in Continuous Gum Work.*

Students should be promptly present at the College on Friday, September 30th, at 10 o'clock A. M., to make the preliminary arrangements for entering upon regular work on the morning of October 1st. Seats in the lecture room are assigned by selection to students in the order of registration on the Steward's books; and each student is expected to occupy during the session such seat as he may select. Students on arriving at Ann Arbor, should call at the Steward's office.

### CONDITIONS OF GRADUATION.

The candidate must be twenty-one years of age. He must furnish evidence of good moral character.

He must devote three years to the study of his profession. He must attend two full courses of lectures in the Dental College, or one course in some college having an equal standard of requirements, and the last one here, and we recommend that he attend three courses regularly.

He must sustain an examination satisfactory to the Faculty in all the branches taught.

A graduate of the Medical College may enter the senior class, and, if found qualified, may graduate after one year has been devoted to the study of dentistry.

### FEES AND EXPENSES.

The fees, which must be paid in advance, are as follows:

RESIDENTS OF MICHIGAN.—Matriculation fee, $10.00; annual dues, $20,00.

NON-RESIDENTS.—Matriculation, $25.00; annual fees, $25.00.

GRADUATION FEE.—For all alike, $10.00. The admission fee is paid but once, and entitles the student to the privileges of permanent membership in any department of the University. The annual due is paid the first year and every year thereafter while at the University.

For further particulars, address the Dean of the Dental College Ann Arbor, Michigan.

**J. TAFT, Dean.**

### THE DENTAL COLLEGE
### OF THE
### UNIVERSITY OF MICHIGAN.

The Seventh Annual Session of this Institution will commence on the 1st of October and close on the last Wednesday in March, thus making a course of six months. The regular course of instruction commences at once, and will proceed through the term, with the customary vacation.

The students in this department will receive instructions in Anatomy, Physiology, Pathology, Chemistry, Materia Medica, Therapeutics and Surgery, from the Professors of their respective branches in the *Department of Medicine and Surgery* of the University, when lectures commence, and continue the same as with the Dental College. There will also, in addition, be a special course upon each of these branches. These courses will each embrace from ten to fifteen lectures.

### FACULTY OF THE DENTAL DEPARTMENT.

J. B. ANGELL, LL. D., - - - - - - - President.
J. TAFT, D. D. S., - Principles and Practice of Operative Dentistry.
CORYDON L. FORD, M. D., D. D. S., - - Anatomy and Physiology.
J. A. WATLING, D. D. S., - - Clinical and Mechanical Dentistry.
W. H. DORRANCE, D. D. S., - Demonstrator of Mechanical Dentistry.

### SPECIAL COURSES.

A. B. PALMER, M. D., - - - - - - Dental Pathology.
DONALD MACLEAN, M. D., - - - - - - Oral Surgery.
E. S. DUNSTER, M. D., Diseases of Women and Children with reference to the Teeth.
GEO. E. FROTHINGHAM, M. D., - - - - Dental Therapeutics.

*Geo L. Field, D. D. S., will give Special Instruction in Continuous Gum Work.*

Students should be promptly present at the College on Friday, September 30th, at 10 o'clock A. M., to make the preliminary arrangements for entering upon regular work on the morning of October 1st. Seats in the lecture room are assigned by selection to students in the order of registration on the Steward's books; and each student is expected to occupy during the session such seat as he may select. Students on arriving at Ann Arbor, should call at the Steward's office.

### CONDITIONS OF GRADUATION.

The candidate must be twenty-one years of age. He must furnish evidence of good moral character.

He must devote three years to the study of his profession. He must attend two full courses of lectures in the Dental College, or one course in some college having an equal standard of requirements, and the last one here, and we recommend that he attend three courses regularly.

He must sustain an examination satisfactory to the Faculty in all the branches taught.

A graduate of the Medical College may enter the senior class, and, if found qualified, may graduate after one year has been devoted to the study of dentistry.

### FEES AND EXPENSES.

The fees, which must be paid in advance, are as follows:

Residents of Michigan.—Matriculation fee, $10.00; annual dues, $20,00.

Non-Residents.—Matriculation, $25.00; annual fees, $25.00.

Graduation Fee.—For all alike, $10.00. The admission fee is paid but once, and entitles the student to the privileges of permanent membership in any department of the University. The annual due is paid the first year and every year thereafter while at the University.

For further particulars, address the Dean of the Dental College, Ann Arbor, Michigan.

**J. TAFT, Dean.**

### OHIO COLLEGE

OF

### DENTAL SURGERY,

**No. 29 College Street, Cincinnati, O.**

THIRTY-SIXTH ANNUAL SESSION, 1881-82.

### FACULTY:

| | |
|---|---|
| JAMES TAYLOR, M. D , D. D. S. | Principles and Practice, and Dental Hygiene. |
| WM. CLENDENIN, M. D. | Anatomy and Surgery. |
| J. S. CASSIDY, M. D., D. D. S. | Chemistry and Materia Oral Medica. |
| H. A. SMITH, D D. S. | Clinical Dentistry. |
| A. O. RAWLS, D. D. S. | Pathology and Therapeutics. |
| J. R. CLAYTON, D. D. S. | Physiology and Histology. |
| FRANK BELL, D. D. S. | Mechanical Dentistry and Metallurgy. |

G. W. KEELY, D. D. S., Lecturer on Causes and Management of Irregularities of the Teeth.

| | |
|---|---|
| C. L. DUDLEY, | Demonstrator of Chemical Analysis |
| H. L. MOORE, D. D. S.<br>J. M. CLYDE, D. D. S. | Demonstrator of Operative Dentistry. |
| WM. KNIGHT, M. D., D. D. S. | Demonstrator of Anatomy. |

This Institution is a DENTAL COLLEGE IN THE STRICTEST SENSE. The property is owned by an Association of Dentists, numbering nearly one hundred. The Faculty is composed of Practitioners of Dentistry, whose purpose it is to give a thorough course of instruction in the theory and practice of Dentistry.

The College Building was designed, erected, and is exclusively used for a Dental School. The reception, lecture, clinical and dissecting rooms, and mechanical laboratory are large, well lighted and admirably arranged for the purposes of dental education. Located in the center of one of our most populous cities, where there is no other Dental School, the supply of patients is abundant, and at times in excess of the requirements for clinical practice. The College Infirmary will be open every afternoon (except Sundays), during the session, thus affording the students ample opportunities to engage in *actual practice* under the direction of the Professors and Demonstrators.

### GRADUATION.

The candidate must be twenty-one years of age. He must have attended two courses of lectures in a Dental or Medical College, the last of which must be in this institution. A reputable dentist in actual practice five years, may be examined for the degree of D. D. S., after attending one full course of lectures. After this session two courses of lectures will be uniformly required for graduation.

### FEES.

| | |
|---|---|
| Matriculation fee (but once) | $ 5 00 |
| Professors' tickets for one session, | 75 00 |
| Dissecting ticket, including material, | 10 00 |
| Demonstrator of Chemical Analysis, | 10 00 |
| Diploma Fee, | 20 00 |

This session begins the 10th of October, and continues until the last of February. Board can be had for from $4.00 to $6.00 per week.

For further information, address

**H. A. SMITH, Dean,**

**286 Race Street, CINCINNATI.**

### OHIO COLLEGE
OF
### DENTAL SURGERY,

**No. 29 College Street, Cincinnati, O.**

THIRTY-SIXTH ANNUAL SESSION, 1881-82.

### FACULTY:

| | |
|---|---|
| James Taylor, M. D., D. D. S. | Principles and Practice, and Dental Hygiene. |
| Wm. Clendenin, M. D. | Anatomy and Surgery. |
| J. S. Cassidy, M. D., D. D. S. | Chemistry and Materia Oral Medica. |
| H. A. Smith, D. D. S. | Clinical Dentistry. |
| A. O. Rawls, D. D. S. | Pathology and Therapeutics. |
| J. R. Clayton, D. D. S. | Physiology and Histology. |
| Frank Bell, D. D. S. | Mechanical Dentistry and Metallurgy. |

G. W. Keely, D. D. S., Lecturer on Causes and Management of Irregularities of the Teeth.

| | |
|---|---|
| C. L. Dudley, | Demonstrator of Chemical Analysis |
| H. L. Moore, D. D. S. } J. M. Clyde, D. D. S. } | Demonstrator of Operative Dentistry. |
| Wm. Knight, M. D., D. D. S. | Demonstrator of Anatomy. |

This Institution is a Dental College in the Strictest Sense. The property is owned by an Association of Dentists, numbering nearly one hundred. The Faculty is composed of Practitioners of Dentistry, whose purpose it is to give a thorough course of instruction in the theory and practice of Dentistry.

The College Building was designed, erected, and is exclusively used for a Dental School. The reception, lecture, clinical and dissecting rooms, and mechanical laboratory are large, well lighted and admirably arranged for the purposes of dental education. Located in the center of one of our most populous cities, where there is no other Dental School, the supply of patients is abundant, and at times in excess of the requirements for clinical practice. The College Infirmary will be open every afternoon (except Sundays), during the session, thus affording the students ample opportunities to engage in *actual practice* under the direction of the Professors and Demonstrators.

### GRADUATION.

The candidate must be twenty-one years of age. He must have attended two courses of lectures in a Dental or Medical College, the last of which must be in this institution. A reputable dentist in actual practice five years, may be examined for the degree of D. D. S., after attending one full course of lectures. After this session two courses of lectures will be uniformly required for graduation.

### FEES.

| | |
|---|---|
| Matriculation fee (but once) | $ 5 00 |
| Professors' tickets for one session, | 75 00 |
| Dissecting ticket, including material, | 10 00 |
| Demonstrator of Chemical Analysis, | 10 00 |
| Diploma Fee, | 20 00 |

This session begins the 10th of October, and continues until the last of February. Board can be had for from $4.00 to $6.00 per week.

For further information, address

**H. A. SMITH, Dean,**
286 Race Street, CINCINNATI.

### OHIO COLLEGE
OF
### DENTAL SURGERY,

**No. 29 College Street, Cincinnati, O.**

THIRTY-SIXTH ANNUAL SESSION, 1881-82.

### FACULTY:

| | |
|---|---|
| JAMES TAYLOR, M. D , D. D. S. | Principles and Practice, and Dental Hygiene. |
| WM. CLENDENIN, M. D. | Anatomy and Surgery. |
| J. S. CASSIDY, M. D., D. D. S. | Chemistry and Materia Oral Medica. |
| H. A. SMITH, D D. S. | Clinical Dentistry. |
| A. O. RAWLS, D. D. S. | Pathology and Therapeutics. |
| J. R. CLAYTON, D. D. S. | Physiology and Histology. |
| FRANK BELL, D. D. S. | Mechanical Dentistry and Metallurgy. |

G. W. KEELY, D. D. S., Lecturer on Causes and Management of Irregularities of the Teeth.

| | |
|---|---|
| C. L. DUDLEY, | Demonstrator of Chemical Analysis |
| H. L. MOORE, D. D. S.<br>J. M. CLYDE, D. D. S. | Demonstrator of Operative Dentistry. |
| WM. KNIGHT, M. D., D. D. S. | Demonstrator of Anatomy. |

This Institution is a DENTAL COLLEGE IN THE STRICTEST SENSE. The property is owned by an Association of Dentists, numbering nearly one hundred. The Faculty is composed of Practitioners of Dentistry, whose purpose it is to give a thorough course of instruction in the theory and practice of Dentistry.

The College Building was designed, erected, and is exclusively used for a Dental School. The reception, lecture, clinical and dissecting rooms, and mechanical laboratory are large, well lighted and admirably arranged for the purposes of dental education. Located in the center of one of our most populous cities, where there is no other Dental School, the supply of patients is abundant, and at times in excess of the requirements for clinical practice. The College Infirmary will be open every afternoon (except Sundays), during the session, thus affording the students ample opportunities to engage in *actual practice* under the direction of the Professors and Demonstrators.

### GRADUATION.

The candidate must be twenty-one years of age. He must have attended two courses of lectures in a Dental or Medical College, the last of which must be in this institution. A reputable dentist in actual practice five years, may be examined for the degree of D. D. S., after attending one full course of lectures. After this session two courses of lectures will be uniformly required for graduation.

### FEES.

| | |
|---|---|
| Matriculation fee (but once) | $ 5 00 |
| Professors' tickets for one session, | 75 00 |
| Dissecting ticket, including material, | 10 00 |
| Demonstrator of Chemical Analysis, | 10 00 |
| Diploma Fee, | 20 00 |

This session begins the 10th of October, and continues until the last of February. Board can be had for from $4.00 to $6.00 per week.

For further information, address

**H. A. SMITH, Dean,**
**286 Race Street, CINCINNATI.**

### OHIO COLLEGE

OF

### DENTAL SURGERY,

### No. 29 College Street, Cincinnati, O.

THIRTY-SIXTH ANNUAL SESSION, 1881-82.

### FACULTY:

| | |
|---|---|
| JAMES TAYLOR, M. D , D. D. S. | Principles and Practice, and Dental Hygiene. |
| WM. CLENDENIN, M. D. | Anatomy and Surgery. |
| J. S. CASSIDY, M. D., D. D. S. | Chemistry and Materia Oral Medica. |
| H. A. SMITH, D D. S. | Clinical Dentistry. |
| A. O. RAWLS, D. D. S. | Pathology and Therapeutics. |
| J. R. CLAYTON, D. D. S. | Physiology and Histology. |
| FRANK BELL, D. D. S. | Mechanical Dentistry and Metallurgy. |

G. W. KEELY, D. D. S., Lecturer on Causes and Management of Irregularities of the Teeth.

| | |
|---|---|
| C. L. DUDLEY, | Demonstrator of Chemical Analysis |
| H. L. MOORE, D. D. S. / J. M. CLYDE, D. D. S. | Demonstrator of Operative Dentistry. |
| WM. KNIGHT, M. D., D. D. S. | Demonstrator of Anatomy. |

This Institution is a DENTAL COLLEGE IN THE STRICTEST SENSE. The property is owned by an Association of Dentists, numbering nearly one hundred. The Faculty is composed of Practitioners of Dentistry, whose purpose it is to give a thorough course of instruction in the theory and practice of Dentistry.

The College Building was designed, erected, and is exclusively used for a Dental School. The reception, lecture, clinical and dissecting rooms, and mechanical laboratory are large, well lighted and admirably arranged for the purposes of dental education. Located in the center of one of our most populous cities, where there is no other Dental School, the supply of patients is abundant, and at times in excess of the requirements for clinical practice. The College Infirmary will be open every afternoon (except Sundays), during the session, thus affording the students ample opportunities to engage in *actual practice* under the direction of the Professors and Demonstrators.

### GRADUATION.

The candidate must be twenty-one years of age. He must have attended two courses of lectures in a Dental or Medical College, the last of which must be in this institution. A reputable dentist in actual practice five years, may be examined for the degree of D. D. S., after attending one full course of lectures. After this session two courses of lectures will be uniformly required for graduation.

### FEES.

| | |
|---|---|
| Matriculation fee (but once) | $ 5 00 |
| Professors' tickets for one session, | 75 00 |
| Dissecting ticket, including material, | 10 00 |
| Demonstrator of Chemical Analysis, | 10 00 |
| Diploma Fee, | 20 00 |

This session begins the 10th of October, and continues until the last of February. Board can be had for from $4.00 to $6.00 per week.

For further information, address

**H. A. SMITH, Dean,**
286 Race Street, CINCINNATI.

### DIRECTORY OF DENTAL SOCIETIES.

*American Dental Association*—Meets on the first Tuesday in August, 1881, in New York. Pres. C, N. Pierce, Philadelphia, Pa.; Cor. Sec. M. H. Webb, Lancaster, Pa.; Rec. Sec- Geo. A. Cushing, Chicago, Ill.

*National Dental Association*—Meets in New York on the second Tuesday of Augus.t 1881. Pres. A. L. Northrop, New York; Sec. R. Finley Hunt, Washington, D. C.

### STATE DENTAL SOCIETIES.

*California State Dental Association*—June 8, 1881. Pres. W. J. Younger, San Francisco; Sec. S. E. Goe., San Francisco; Cor. Sec. H. E. Knox, San Francisco.

*Connecticut State Dental Society*—Pres. E. T. Payne; Rec. Sec. Geo. L. Parmelee; Hartford. Semi-annual meeting in October, 1880, at Hartford.

*Dental Society of the State of Maryland and District of Columbia*—Meets annually. Next meeting at Washington City, on the second Tuesday in October, 1880. Pres. B. F. Coy, of Baltimore; Sec. M. W. Foster, M. D.

*Georgia State Dental Society*—Meets second Monday in July, 1881, at Augusta. Pres. W. C. Wardlow, Augusta, Rec. Sec. R. A. Holliday, Atlanta, Cor. Sec. L. D. Carpenter, Atlanta.

*Illinois State Dental Society*—meets annually. Pres. C. A. Kitchen, Rockford; Sec E. Noyes, Chicago. Next place of meeting, Bloomington, second Tuesday in May, 1881.

*Indiana State Dental Society*—Meets next year at Indianapolis, on the last Tuesday of June, 1881. Pres. Robert Van Valzah, Terre Haute; Sec. W. N. Hall, Terre Haute.

*Iowa State Dental Society*—Meets annually. Next meeting at Oskaloosa, on the first Tuesday after the 4th of July, 1881. Pres. W. P. Dickinson, Dubuque; Rec. Sec. E. E. Hughes, of Newton; Cor. Sec. L. C. Ingersoll, Keokuk.

*Kentucky State Dental Association*—Meets annually, first Tuesday in June, 1881. Next meeting in Lexington. Pres. C. G. Edwards, Louisville; Sec. W. W. Justice, Winchester.

*Kansas State Dental Association*—Pres. A. Doud, Sec. R. J. Pearson, of Atchison: Next meeting will be held at Emporia, first Tuesday in May, 1881.

*Maine Dental Society*—Meets in August, at Thomaston. Pres. E. J. Robert, Agusta, Sec. C. E. Hussey, Beddeford.

*Massachusetts Dental Society*—Meets semi-annually. Pres. J. H. Kidder, of Lawrence; Rec. Sec. Dwight M. Clapp, Boston; Cor. Sec. T. H. Chandler, Boston.

*Michigan State Dental Association*—Meets annually. Next meeting at Detroit, March 31, 1881. Pres. J. C. Parker, Grand Rapids. Sec. M. F. Finley, Ypsilanti. Treas. J. Lathrop, Detroit.

*Mississippi State Dental Association*—Will meet third Wednesday of September, 1880 at Jackson, Pres. A. H. Hilzheim, Jackson; Sec, M. C. Marshall, Winona.

*Minnesota Dental Association*—Meets annually. Meets May 26, 1881, at Winona. Pres. W. F. Lewis, Sec. C. M. Bailey, Minneapolis.

*Missouri Dental Association*—Meets annually on the first Tuesday of June, 1881 Next meeting at Sweet Springs. Pres. A. N. Fuller; Sec. W. H. Eames, St. Louis.

*Nebraska State Dental Society*—Meets annually. Next meeting July 23, 1881, at Fremont. Pres. J. W. Chadduck, Nebraska City; Sec. W. F. Roseman, Fremont,

*New Jersey State Dental Society*—Meets annually. Next meeting at Long Branch. on Wednesday, July, 20th 1881. Pres. F. A. Levey, Orange; Sec. C. A. Meeker, Newark.

*New Hampshire Dental Society*—Pres. D. W. Eagerly, Farmington; Sec. C. W. Clerment, Manchester.

*North Carolina State Dental Society*—Meets annually. Next meeting at Raleigh, first Wednesday in September, 1880. Pres. V. E. Turner; Sec. D. A. Robinson.

*Ohio State Dental Society*—Meets annually at Columbus, first Tuesday of December, 1880. Pres. C. H. Harroun, Toledo; Rec. Sec. W. H. Sillito, Xenia; Cor. Sec.

*Oregon State Dental Society*—Meets annually. Pres. J. R. Cardwell, Portland; Rec. Sec. S. J. Barber.

*Pennsylvania State Dental Society*—Meets Tuesday, July 27th, 1881, at Bellefonte. Pres. H. Gerhart, Lewisburg; Rec. Sec. E. P. Kremer, Lebanon, Pa.; Cor. Sec. E. B. Long, Pittston.

*Rhode Island Dental Association*—Pres. A. W. Buckland, Woonsocket; Sec. L. L. Buckland, Providence.

*South Carolina State Dental Association*—Meets annually. Pres. B. A. Muckenfuss; Sec. G. F. S. Wright, Columbia; Cor. Sec. B. H. Teagne, Aikin. Next meeting at Charleston, the second Tuesday of July, 1881.

*Tennessee Dental Association*—Meets Semi-annually. Next meeting at Nashville, fourth Wednesday in June, 1881. Pres. A. Bartman; Rec. Sec. L. G. Noel, Nashville; Cor. Sec. W. H. P. Jones.

*Vermont State Dental Society*—Meets annually. Pres. J. L. Perkins, St. Johnsburg; Sec. C. D. Newell, St. Johnsburg.

*Virginia State Dental Association*—Meets in Charlottsville, Tuesday, August 19, 1880 Pres. J. Hal Moore; Sec. L. M. Cowarden, Richmond.

*Wisconsin State Dental Association*—Meets annually. Next meeting atMilwaukee, July 20, 1881. Pres. M. T. Moore, La Crosse; Sec. Geo. H. McCansey, Janesville.

*The Dental Society of the State of New York*—Meets annually on the second Wednesday in May. Next session at Albany, May 12, 1881. Pres. C. E. Francis, New York; Rec. Sec. S. A. Freeman, Buffalo; Cor. Sec. W. H. Atkinson, New York.

### LOCAL SOCIETIES.

*American Academy of Dental Science*—Meets annually in Boston, Mass., on the last Wednesday in October, 1880. Pres. E. G. Tucker; Rec. Sec. E. P. Bradbury; Cor. Sec. C. P. Wilson, Boston.

*Brooklyn Dental Society*—Meets monthly. Pres. Chas. D. Cooke; Cor. Sec. W. H. Atkinsyn. Rec. Sec. A. P. Crandall, 508 Clinton Ave., Brooklyn.

*Connecticut Valley Dental Association*—Meets June 17, 1881, at Savin Rock Conn. Pres. C. T. Stockwell, Springfield, Mass. Sec. A. M. Ross, Chicopee.

*East Tennessee Dental Association*—Meets annually. Next meeting will be held at Chattanooga, Tenn., on the first Wednesday of Aug., 1880. Pres. S. M. Prothro, Chattanooga; Sec. A. W. Palmer, Kingston.

*Merrimack Valley Dental Association*—Meets annually. Pres. J. N. Chandler, Boston; Sec. H. W. Coburn, Lowell, Mass.

*Mississippi Valley Dental Association*—Meets annually at Cincinnati, on first Wednesday in March, 1881. Pres. H. M. Reid, Cincinnati; Sec. E. G. Betty, Cincinnati.

*Nashville Dental Association*—Meets on the second Tuesday of each month, Pres. R. R. Freeman; Sec. L. G. Noel, Nashville.

*Northern Ohio Dental Association*—Meets annually. Next meeting at Akron, on the second Tuesday in May, 1881. Pres. J. W. Lyder, Akron, O; Cor. Sec. H. F. Harvey, Cleveland.

*Ohio College Dental Association*—Meets annually, Tuesday, March 2, 1881, at Cinc nati. Pres. F. A. Hunter, Cincinnati; Rec. Sec. H. A. Smith, Cincinnati.

*Odontographic Society of Pennsylvania*—Meets on the first Wednesday of each month, at 8 o'clock p. m., in the Philadelphia Dental College. Pres. J. L. Eisenbrey, Rec. Sec. E. L. Hewitt.

*Odontological Society of New York City*—Meets the second Tuesday of each month. Pres. W. A. Bronson; Rec. Sec. Wm. Jarvie, Jr.

*Pennsylvania Association of Dental Surgeons.*—Pres. J. H. Githens. Rec. Sec. Theo. F. Chupein.

*Pittsburg Dental Society*—Meets the second Tuesday Evening of each month. Pres. N. W. Aithen; Sec. L. Depriz.

*Fifth District Dental Society of N. Y.*—Meets semi-annually. Next meeting in Syracuse, on the 12th of October, next. Pres. E. L. Swartwout, of Utica. Sec. I. C. Curtis of Fulton.

### EXCHANGES.

St. Louis, Medical and Surgical Journal.
The Pacific Medical and Surgical Journal, San Francisco.
Cincinnati Medical Advance.
Cincinnati Lancet and Clinic.
Dental Cosmos, Philadelphia.
Ladies' Repository, Cincinnati.
Chicago Medical Examiner.
The Detroit Review of Medicine and Pharmacy.
Medical Record, N. Y.
Nashville Journal of Medicine and Surgery.
American Journal of Dental Science, Baltimore.
The United States Medical and Surgical Journal, Chicago.
Canada Journal of Dental Science, Montreal.
Indiana Journal of Medicine.
Missouri Dental Journal, St. Louis.
The Sanitaran, New York City.
The Monthly Review of Dental Surgery, London.
L'art Dentaire, Paris.
Le Progress Dentaire, Paris.
Deutische Verteljahrsschrift, Leipzig.
Physician and Surgeon, Ann Arbor, Mich.

### CONTENTS.

### COMMUNICATIONS.

Treatment of Pulpless Teeth and Manner of Filling Roots.............. 447
Operative Dentistry.............................................................. 489
The Treatment of Pulpless Teeth.......................................... 491
The Merits of Soft and Cohesive Gold as Filling Materials............. 493
What Ails the Dental Societies?.............................................. 497

### SELECTIONS.

Medical Students.................................................................. 499

Our Medical Literature.......................................................... 503

Rodent Ulcer and Epithelioma................................................. 511

Practical Hints on Mounting.................................................... 512

The Compensation of Expert Witnesses..................................... 513

Deaths from Ether................................................................ 514

Fatal Poisoning by Inhalation of Carbolic Acid............................ 515

Hints for Using Iodoform........................................................ 515

The Ventilation of Bed Rooms.................................................. 516

Treatment of Nasal Catarrh..................................................... 517

Action Upon the Fœtus of Medicine taken by the Mother................ 518

Claims of the Microscope........................................................ 519

The Care of the Dentists' Hands.............................................. 520

The Hippocratic Oath............................................................. 521

Curious Sanitary Discoveries................................................... 522

Mouth Wash for Tobacco Consumers......................................... 523

### EDITORIAL.

Engagement Books................................................................ 523

The Physicians' Visiting List.................................................. 523

The Dental Law in Ohio.......................................................... 524

Ohio State Society................................................................ 524

Universal Rubber Dam Screw-Clamp.......................................... 525

Rubber Dam Holder............................................................... 526

Removal............................................................................... 526

---

### THE DENTAL REGISTER,

A MONTHLY MAGAZINE DEVOTED TO THE INTERESTS OF

**THE DENTAL PROFESSION.**

*TERMS REDUCED TO $2.50 PER YEAR. SINGLE COPIES 25C.*

EDITED BY PROF. J. TAFT, D.D.S.

AND

*Published by SPENCER, CROCKER & CO., Ohio Dental Depot.*

The volume commences in January and closes in December.

The REGISTER being issued monthly is always fresh, therefore Indispensable to the practitioner who desires to keep posted up with the new inventions and improvements which are daily developed. Neither expense nor effort will be spared to give value and variety to its contents. Articles will be illustrated with well executed engravings whenever they will add to the interest or assist to explanation.

Its extensive circulation and frequency of issue make it one of the BEST DENTAL ADVERTISING MEDIUMS in the United States.

All subscriptions must begin with January or July.

Local or special notices, five lines or less, will be inserted for $3 each insertion; over five lines, 50 cts. per line.

All advertising bills payable in advance, or at furthest, after the issue of the first number containing the advertisement. All transient advertisements to be paid for in advance.

☞CONTRIBUTIONS SOLICITED.

Exclusively Editorial Communications should be addressed to Editor.

The latest number of the REGISTER may be ordered with or without other goods at any time, and all letters should be addressed to

*SPENCER, CROCKER & CO, Ohio Dental Depot, Cincinnati, O.*

